# Exploring the Nature of Desmosomal Cadherin Associations in 3D

**DOI:** 10.1155/2010/930401

**Published:** 2010-06-21

**Authors:** Gethin R. Owen, David L. Stokes

**Affiliations:** ^1^New York Structural Biology Center, 89 Convent Avenue, New York, NY 10027, USA; ^2^Department of Cell Biology, Skirball Institute of Biomolecular Medicine, New York University School of Medicine, New York, NY 10016, USA

## Abstract

Desmosomes are a complex assembly of protein molecules that mediate adhesion between adjacent cells. Desmosome composition is well established and spatial relationships between components have been identified. Intercellular cell-cell adhesion is created by the interaction of extracellular domains of desmosomal cadherins, namely, desmocollins and desmogleins. High-resolution methods have provided insight into the structural interactions between cadherins. However, there is a lack of understanding about the architecture of the intact desmosomes and the physical principles behind their adhesive strength are unclear. Electron Tomography (ET) studies have offered three-dimensional visual data of desmosomal cadherin associations at molecular resolution. This review discusses the merits of two cadherin association models represented using ET. We discuss the possible role of sample preparation on the structural differences seen between models and the possibility of adaptive changes in the structure as a direct consequence of mechanical stress and stratification.

## 1. The Desmosome-a Historical Perspective

The desmosome ultrastructure has been the topic of many investigations since it was first described by the Italian pathologist Bizzozero in 1864 [1]. Schaffer [2] later introduced the term “desmosome” from the greek “desmos” meaning bond and “soma” meaning body. During this era the desmosome was thought to be a cytoplasm-filled intercellular bridge. This hypothesis was dismissed by the electron microscopy (EM) study of desmosomes by Porter [3], which was the first to display the desmosome as contacts between adjacent cells. A novel staining approach for EM by Rayns and coworkers [4] suggested that discrete dense particles of 4 nm in diameter provided the connections between cells. These particles were arranged in a staggered pattern with respect to the opposing membrane establishing a zipper-like mechanism of intercellular adhesion. Later on in the 1970's ultrastructural work focused on desmosome formation [5] and was complemented by biochemical and molecular biology approaches. The latter techniques were applied to identify the desmosome components and characterize their interactions [6–10] enabling the production of antibodies against each component. The spatial relationships between individual desmosome components were then identified, at the EM level, by immunogold localization [11]. Once the individual components involved in intercellular adhesion were established, high resolution EM [12], X-ray crystallography [13], Nuclear Magnetic Resonance (NMR) [14], and molecular force approaches [15] were used to study the extracellular associations between transmembrane glycoproteins named desmosomal cadherins, thus shedding light on the ability of desmosomes to resist mechanical shear force.

At present the main focus of investigations involve the role of desmosomes in cell differentiation [16–18], cancer [19] and inherited diseases of the heart and skin [20]. However, a revived interest in the structural mechanism of desmosomal function has become more prominent in the last few years with the advent of electron tomography (ET). ET provides high-resolution imaging of cellular complexes in their natural cellular environment, thus bridging the gap between structural studies at the single molecule (NMR, X-ray crystallography) and cellular (Transmission Electron Microscopy (TEM), Light Microscopy) levels. ET studies have offered three-dimensional visual data of desmosomal cadherin associations at molecular resolution [21–23] complementing biochemical and molecular data. However, differences have been observed in the organization of the desmosomal cadherins, raising questions about the preservation of native structure during specimen preparation. 

The purpose of this review is to discuss (1) the desmosome structure and its importance in maintaining tissue integrity; (2) high-resolution models of cadherin associations; and (3) current ET models of desmosomal cadherin associations *in situ*.

## 2. Desmosome Structure and Tissue Integrity

Today, desmosomes are widely recognized as an adhesive junction that are prevalent in tissue subjected to shear force (e.g., skin and heart and many epithelia). Desmosomes maintain tissue organization and provide mechanical strength by linking intermediate filaments (IF) networks of neighboring cells to each other, thus producing a scaffold that propagates across the entire tissue [24]. 

**Figure 1:**
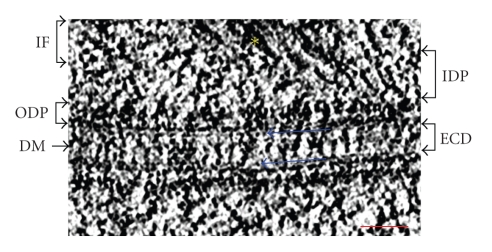
A tomographic slice of a desmosome bridging two opposing keratinocytes (delineated by two blue arrows) from the epidermis of a newborn mouse. Intermediate filaments (labeled IF and marked by a yellow asterix) from neighboring cells are interconnected to each other via the transmembrane cadherin proteins. Intracellular bridging proteins of the inner dense plaque (labeled IDP is a structural framework that includes cadherin intracellular domains, plakoglobin, plakophillin, and the N-terminal domain of desmoplakin) and the outer dense plaque (labeled ODP is a structural framework consisting of the rod domain and C-terminal domain of desmoplakin) link the intermediate filaments to the extracellular core domain (ECD). The ECD is composed of extracellular domains of desmosomal cadherins, namely, desmocollins and desmogleins. The area of cadherin interaction appears as a dense mid-line bisecting the ECD due to heterophilic associations between desmocollins and desmogleins. Scale bar represents 50 nm.

Structurally desmosomes resemble rivets at cell borders with a diameter of approximately 300 nm. They have two principal domains: the extracellular core domain (ECD) or “desmoglea” which is ~30 nm wide and bisected by a dense mid-line region, and a cytoplasmic dense plaque which lies parallel to the plasma membrane and separated from it by a less dense zone. The ECD is made up of extracellular domains of transmembrane glycoproteins belonging to the cadherin super family, which generally mediate calcium-dependent cell-cell adhesion in vertebrate tissue [25–27]. The desmosomal cadherins are called desmocollin (Dsc) and desmoglein (Dsg) and their cytoplasmic tails bind to a heterogeneous assembly of cytoplasmic components (desmoplakin (DP) I and II, plakoglobin (PG) and plakophilin (PKP)) to form the cytoplasmic dense plaque at the intracellular surface of the membrane. By providing the anchoring site for IF, this plaque indirectly connects the intercellular cadherins to the IF network of the cell (Figure 1).

Disruption of the desmosome structure generally has devastating consequences to the integrity of the tissue and the viability of the organism. For example, knockout of Dsc-1 causes epidermal fragility, barrier defects, abnormal differentiation, hyperproliferation, and hair loss [28] whereas the knockout of Dsc-3 causes permeability barrier defects of the stratum corneum and mice die shortly after birth with severe dehydration [29]. The targeted Dsc-3 null mutation is lethal before implantation in homozygous mutants [30] whereas the targeted Dsg-2 null mutation is lethal shortly after implantation. In the case of Dsg-2, defects are thought to be desmosome independent during early development when Dsg-2 is needed for survival of both embryonic stem cells and the early embryo [31]. 

The importance of cytoplasmic plaque proteins such as DP or PG is also evident from mutational studies. Dsp mutant embryos die very early in E5.5–6.5 owing to defects in the extraembryonic tissues causing the expansion failure of the developing embryo egg cylinder [32]. Most PG-null embryos die of heart failure from E10.5–12.5 onwards [33]. However those PG-null mice that survive birth exhibit skin blistering and heart abnormalities [34]. Mutations to the desmosome components, such as DP and PG found in human genetic diseases, can lead to heart [16], skin [35], and hair defects [36]. Even Dsg-2 mis-expression has been associated with human squamous cell carcinomas for example, gastric cancer, where it is underexpressed, overexpressed [37] or upregulated [38]. However, the dependence of tissues on desmosome integrity is most evident in the autoimmune disease pemphigus vulgaris [39] and the exfoliative toxin in staphylococcal scalded skin syndrome [40]. Pemphigus vulgaris gives rise to acantholysis (i.e., the loss of cell-cell adhesion between keratinocytes) due to binding of autoantibodies to the EC1 domain of Dsg-1 and Dsg-3 [41], thus inhibiting cell-cell adhesion through steric hindrance [42]. In staphylococcal scalded skin syndrome superficial epidermal splitting is caused by the exfoliative toxin serine protease, which cleaves Dsg-1 between EC3 and EC4 again disrupting the cell-cell adhesion between keratinocytes [43]. Recent evidence suggests that antibody binding to desmosomal cadherins trigger external cascades, which may indeed amplify the disease pathogenesis [44] supporting the premise that the desmosome is a dynamic rather than a static structure.

## 3. High-Resolution Models of Cadherin Associations

When first formed, desmosome cadherin associations are relatively weak, but eventually they are capable of locking themselves in a hyperadhesive state [45]. Unlike the early desmosomes, this hyperadhesive junction is resistant to disruption by the calcium chelator, ethylene glycol bis (2-aminoethyl ether)-N,N,N′,N′- tetraacetic acid (EGTA) and is termed calcium independent [45]. In wound healing situations it has been demonstrated that desmosomes can revert to the weaker, calcium dependent state, when cell migration is necessary for regeneration of the epithelium. These observations indicate that the desmosome is not a static entity and is able to respond to environmental cues. The mechanism for controlling these adhesive states is believed to involve protein kinase C signaling, directly affecting cadherin association in the ECD [46]. Although no high-resolution structural work on desmsomes has yet addressed these states, there is evidence that cadherin organization may be involved. Understanding the mode of cadherin associations in these adhesive states would be a significant step in determining how signaling can alter adhesivity and would provide possible targets for treating conditions related to compromised desmosome integrity.

**Figure 2:**
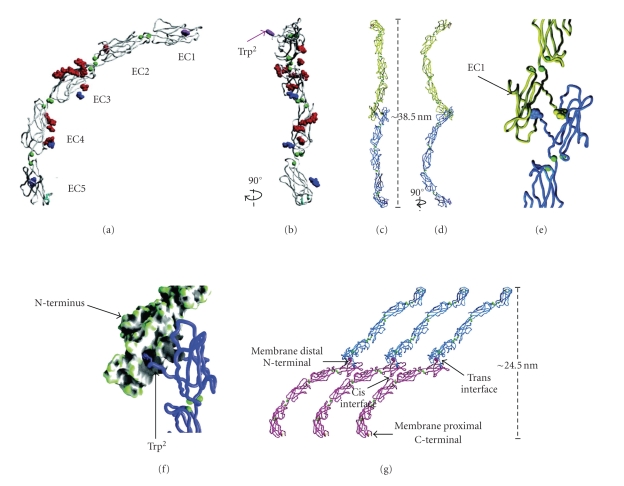
Type I or classical cadherin structure obtained from X-ray crystallographic studies. Structurally the extracellular portion of the cadherin molecule (also known as ectodomains and abbreviated EC) appears as five tandem Ig-like domains, each domain adopting a *β*-sandwich fold with the topology of a Greek key. Between each domain reside three calcium ions (green spheres) conferring rigidity to the domain interface and inducing the entire structure to assume an extended curve with a defined tilt of ~100° between the long axes of EC1, and 5. Disulfide bonds, O-linked sugars, and N-linked sugars are represented in cyan, red, and blue, respectively (a). In classical cadherins a conserved tryptophan residue near the N-terminus of EC1 has been shown to be crucial in the process of adhesion and is also conserved in desmosomal cadherins (abbreviated to Trp^2^ and is depicted in purple by space filling representation in both a, and b. This Trp^2^ is visibly protruding from the cadherin molecule when viewed at 90° rotation from a (b)) The tip-to-tip associations between the EC1 domains of cadherin molecules from opposing cells membranes form the strand dimer interface (c and at 90° rotation in d). EC1 domain binding interaction between opposing cadherins is highlighted in the magnified view (e). The side chain of Trp^2^ has been suggested as a possible binding site within a hydrophobic pocket in EC1 of a corresponding partner cadherin from the opposing cell membrane. Trp^2^ side chains (displayed as a backbone worm trace) can be seen inserting into a large concave cavity (depicted as grey shading in the molecular surface model) of the opposing EC1 domain (f). As the Trp^2^ is conserved in desmosomal cadherins it is assumed that the Trp^2^ interaction from classical cadherin studies is a compelling model for desmosomal cadherin association, forming a symmetric interdigitation interaction between cadherins of opposing cell membranes, and is known as the strand dimer model (g). Cadherin ectodomains from opposing cells are represented by blue or pink colors. Trp^2^ side chains (space filling representation) are depicted in the color representing the cell. Yellow represents disulfide bonds. Modified from [50] reprinted with permission from AAAS.

Cadherin association in the ECD is a calcium dependent process and defines this family of adhesion molecules. Cadherins are composed of an extracellular portion with five tandem Ig-like domains (Figures 2(a) and 2(b)), a single transmembrane helix and a cytosolic domain designed to interact with various proteins that compose the intracellular plaque. Each extracellular domain consists of ~110 amino acids [47] that adopt a *β*-sandwich fold with the topology of a Greek key [14, 48] of which the dimensions are 4.5 × 2.5 × 2.5 nm^3^. Three calcium ions are bound to the loop connecting successive domains via conserved sequence motifs [49, 50]. X-ray crystallographic data reveal that the three calcium ions bind between successive domains and are coordinated by conserved amino acids at the base of one domain and the top of the next and this coordination is similar in each of the four interdomain interfaces. In this way calcium binding confers rigidity to the domain interface and has been shown to induce extension of the entire extracellular portion of the molecule [12]. In the fully extended state, the molecule assumes a curved structure [51] with an angle of ~100° between the N-terminal EC1 domain and the juxtamembrane EC5 domain [50]. 

**Figure 3:**
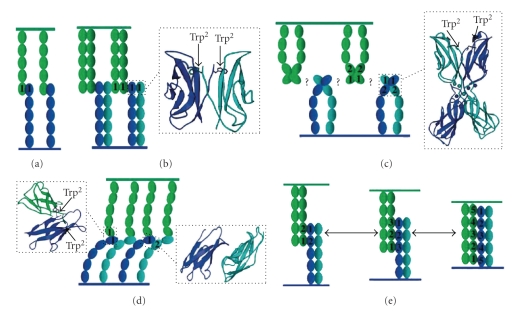
A visual representation of alternative desmosomal cadherin association models from the strand dimer (a), that consider the heterophilic interactions of desmosomal cadherins. Individual desmosomal cadherin ectodomains (1 to 5) are portrayed as ovals and are colored accordingly to clearly separate lateral from strand interactions. Identical color-coding is used to separate lateral and strand interactions in the ribbon representations. Cadherin ectodomains spanning from one cell surface are colored dark or light blue respectively and ectodomains spanning from an opposing cell surface are shown in green. (b) Cadherin dimerization via the surface of EC1 domains containing His^233^/Val^235^ residues (residues not shown). Lateral dimers interact with strand dimers from an opposing cell. The insert shows a ribbon representation of the Trp^2^-mediated lateral-contact. (c) Lateral dimerization via EC1/EC2 calcium binding sites facilitated by the rod-like shape of cadherin ectodomains. The inset shows a ribbon representation of the domain pair dimer from the same cell surface, the position of the calcium ions are represented by spheres and are color coded dark or light blue to represent the cadherin ectodomains in c. (d) Lateral interaction at EC1 between strand dimers bound together by EC1 and EC2 interactions. The left inset is a ribbon representation of Trp^2^ mediated contact between N-terminal domains; the right inset is a ribbon representation of lateral interactions between EC1 and EC2 domains. (e) Adhesive interactions between antiparallel cadherin molecules along their full length. Strand interactions occur between more than two EC domains resulting in the formation of three types of adhesive complexes of different length. Modified from [53] with permission from Birkhäuser Verlag AG.

The majority of the structural studies on cadherin-cadherin interactions have been carried out on the classical cadherins or type I cadherins. A conserved tryptophan residue near the N-terminus has been shown to be critical for adhesion in this type of cadherin (Figures 2(c) and 2(d)). The side chain of Trp^2^ has been suggested to bind within a hydrophobic pocket in EC1 of a crystallographic neighbor, presumably corresponding to a partner cadherin from the opposing cell membrane [13, 48, 50] (Figure 2(e)). This interaction is known as the strand dimer (Figure 2(f)). Although desmosomal cadherins engage in heterophilic interactions whereas classic cadherins engage in homophilic interactions, interestingly despite their functional distinctions, Trp^2^ is also conserved in desmosomal cadherins and is believed to be critical for heterophilic interactions. Heterophilic interactions can be formed by strand (otherwise known as cis) or lateral (otherwise known as trans) interactions. Lateral interactions describe cadherin domain associations from the same cell surface whereas strand interactions describe cadherin domain associations between opposite cell surfaces. Both lateral, trans, strand and cis interactions will be used in this review in accordance to the terms used in the original work. Due to the greater binding partner options available to desmosomal cadherins alternative models to the strand dimer model (Figure 3(a)) have been suggested as seen in Figures 3(b)–3(e). These being as follows.

1. Cadherin adhesive dimerization by both lateral and strand associations via the surface of EC1 domain containing His^233^/Val^235^ residues [13] (Figure 3(b)).
2. Lateral dimerization via the EC1/EC2 calcium binding sites [49, 51] (Figure 3(c)).
3. Lateral interaction between the EC1 and EC2 domains [50] (Figure 3(d)).
4. Adhesive interactions between antiparallel cadherin molecules along their full length-interdigitation model [52] (Figure 3(e)).

The strand dimer and the interdigitation models are the most plausible candidates for cadherin heterophilic interactions. This is because there is no evidence for adhesive interactions via the His^233^/Val^235^ containing EC1 domain surface or for lateral dimerization via the EC1–EC2 calcium-binding site [54–57]. However, the strand dimer model emphasizes symmetric interaction between EC1 domains and the interdigitation model implies very different interactions of EC1 with EC2–EC5. A recent study using intramolecular force microscopy may resolve the discrepancies between the two models [58]. This study confirms the existence of multiple binding sites for cadherins and proposes that as cadherins are pushed together, multiple bonds are formed as they form a parallel alignment in the middle of the extracellular gap. An increase in the curvature in the EC2-4 domains would be required to accommodate this parallel interaction at the mid-point between cells allowing for the formation of a mid-line bisecting the ECD [59]. Such cadherin associations would allow for the domain swap between EC1 domain proposed by Shapiro et al. [13] and Boggon et al. [50] and could explain the existence of associations between other extracellular domains proposed Zhu et al. [52].

## 4. Electron Tomography of Desmosomal Cadherin Associations

The models, described in the previous section, provide evidence for possible modes of desmosomal cadherin associations. However, an explanation for the ability of cadherins to recognize specific binding partners is still unresolved. The difficulty lies in the artificial molecular constructs required for NMR or X-ray crystallography. In contrast, ET allows the study of desmosome structure in the native cellular environment.

Over the past decade technical advances in TEM hardware, imaging software, and sample preparation methods have significantly increased the resolving power of such instruments. The technique of ET has gained popularity as a high resolution imaging method because it provides three-dimensional visual data from any given asymmetric object and bridges the gap between structural studies at the molecular level and the cellular level [60]. Most importantly ET allows the investigation of the molecular architecture of cellular complexes in their natural cellular environment and therefore can be used to elucidate their structure-function relationships. Currently, a significant amount of data investigating desmosomal cadherin associations has been collected by ET. Before elaborating on the findings we will briefly introduce the concept of ET and the methodology involved. This understanding should then allow the reader to follow the discussion on the differences seen in each desmosomal cadherin association model and the possible reasons for those differences.

### 4.1. Electron Tomography

The principle of creating a three-dimensional (3D) object from two-dimensional (2D) projections was conceived by Radon [61]. Theoretical predictions were then put into practice much later by DeRosier [60] when computing technology became available. The steps of creating a 3D object involve collecting 2D projection images at different tilt angles. The 2D projections are then represented as a 2D Fourier transform, which corresponds to the central section of the objects' 3D Fourier transform, that is, normal to the imaging angle. By tilting the object to different angles, the objects' entire Fourier transform can conceivably be sampled. The resolution of the technique is dependent on range and increment of the tilt angles. Due to practical limits of using a planar sample, the tilt range is limited to 160°, whereas the tilt increment is dependent on the electron dose that can be tolerated by the sample. Practically, the resolution of the technique depends upon the method of sample preservation and data collection parameters [62]. These two points will be briefly discussed in the next section.

### 4.2. Sample Preservation

Water is important in biological samples because the surfaces of nearly all biological macromolecules, macromolecular assemblies, and biological membranes are hydrophilic. Due to the nature of the exposed chemical groups transient hydrogen bonds are formed with water, forming a hydration shell. This shell prevents molecules from aggregating and maintains protein conformation. For this reason, native biological samples are incompatible with the high vacuum conditions maintained in the EM and are generally dehydrated prior to being embedded in a hard plastic and sectioned. 

This process is innately destructive to the components of the tissue and a major goal of specimen preparation is to preserve cellular structure at a scale finer than the desired resolution. Preserving the specimen with cross-linking chemicals helps it withstand aggregation and extraction during dehydration. However, the conformation of the molecular constituents are often affected by this fixation and do not represent the native state. 

Cryofixation is an alternative that exploits the high water content in cells and utilizes it as a physical fixative. Theoretically, it has the potential to preserve biological structures at the atomic level. Amorphous ice, a non-crystalline vitreous state of water, is the goal of cryofixation. Two techniques are currently used to cryofix samples for ET and are chosen depending on the size of the sample. High pressure freezing (HPF) is used to preserve bulk samples. This technique subjects the sample to a pressure of 2045 bar, thus decreasing the melting point of water to a minimum of 251 K. Within a few milliseconds a jet of liquid nitrogen cools the sample below the vitrification temperature. Plunge freezing is the technique normally used for smaller samples normally present in an aqueous suspension. Samples in aqueous suspension are spread thinly onto a glow discharged holey carbon grid and plunged into a cryogen at 1 m/s, embedding them in vitrified ice. Samples preserved with both methodologies can be imaged in their native frozen state at low temperature. Both methodologies serve a purpose in cryofixation, namely, to preserved samples of differing sizes. Samples preserved in vitrified thin films created by plunge freezing are generally isolated macromolecular complexes that are small enough to fit in a 200–300 nm thick layer of water. Objects larger than 1 *μ*m are too thick to be observed by normal TEM and also too thick to vitrify by plunge freezing. Both problems can be overcome by HPF followed by physically cutting the vitrified sample into thin sections that can be observed with the TEM. This method is known as cryoelectron microscopy of vitreous sections (CEMOVIS) and has been shown to be applicable for tissue preservation in electron tomography [63]. Although cryoultramicrotomes are commercially available for cutting these sections, practical difficulties in handling these thin flakes of tissue and, specifically, in establishing their adherence to EM specimen support films make this method very technically demanding and its application is therefore limited to a few specialized laboratories worldwide.

An alternative technique known as freeze substitution has been developed so that cryofixed specimens can be imaged at room temperature with minimal changes from the vitrified state. The process of freeze substitution involves substituting the amorphous ice produced by HPF with solvents while maintaining the sample in the vitrified (frozen) state. Fixatives can optionally be added to the solvent so that cell constituents are preserved as the hydration shell is slowly removed by the dehydrating action of the solvent. Such mild conditions for fixation and dehydration minimize artifacts associated with aggregation and extraction. When the sample eventually reaches room temperature, after a step-wise increase in temperature, it is dehydrated and can then be embedded in epoxy resin and processed for imaging.

### 4.3. Data Acquisition

Whether the goal is to image the biological sample embedded in vitreous ice or in epoxy resin the irradiation of both samples by electrons can damage the specimen and alter the structures of interest. Low dose microscopy is a technique used during ET data acquisition to minimize the electron dose on the specimen while maximizing the signal-to-noise ratio in the resulting image [64]. 

### 4.4. In Situ Desmosome Cadherin Association Models in 3D

Desmosomes are readily identifiable in 2D TEM micrographs by their lamellar structure, prominent dense mid-line between opposing cell surfaces, and intermediate filaments inserting into an electron dense cytoplasmic plaque. Only two models of desmosomal cadherin associations in 3D have been described and their findings remain controversial. In the next section each model will be described and their differences discussed.

**Figure 4:**
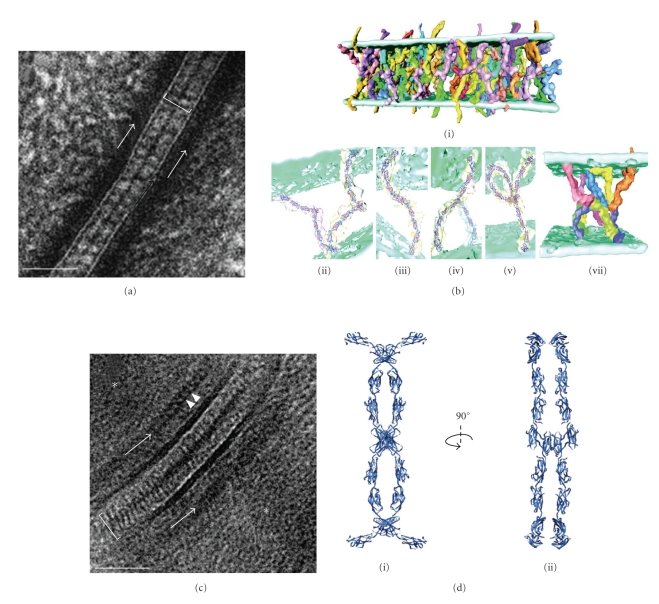
Two models of desmosomal cadherin associations generated from two methods of preparation. A single TEM projection of a newborn mouse epidermis desmosome preserved by high pressure freezing followed by freeze substitution (a). The dense midline is visible bisecting the extracellular core domain (ECD) (white line). Cadherins spanning the extracellular space are separated in an irregular nonsymetrical fashion. A model of cadherin association generated from a dual axis tomogram of newborn mouse epidermis (b i) preserved as in a. C-cadherin crystal structure has been traced onto densities in the ECD believed to be cadherins. The model displays the irregularity of cadherin associations however a dense mid-line is created by tip-to-tip association. The arrangement of cadherins differs to that of the strand dimer model. Three distinct geometries were found resembling the letters W (b ii), S (b iii), and the greek letter *λ* (b iv). Trans interactions mostly occurred in W and S whereas with *λ*, cis interactions mostly with S occurred if a third molecule was added (b v). An addition of a third molecule in cis could form a network in associations between up to 6 molecules (b vi). A single TEM projection of a desmosome from vitrified human epidermis processed by cryoelectron microscopy of vitreous sections (c). Densely packed and periodically arranged cadherins can be seen spanning the extracellular space in a straight manner (white line) and associating to form a dense mid-line. A model of cadherin association generated from a single axis tomogram of vitrified human epidermis processed as in c (d). The ribbon representation of the cadherin association model shows a periodic arrangement of curved cadherin densities that are organized with alternating trans and cis associations tip-to-tip. Two distinct geometries were visible in the model. The W shape formed the trans interactions (see d i) whereas the V-shape formed cis interactions resulting in a highly packed regular zipper-like organization. The organization is better visualized when then model is rotated at 90° from d i, revealing two trans interactions and one cis interaction (d ii). Images 4 B modified from [21] reprinted with permission from AAAS. Images 4 a & c modified from, [65] reprinted with permission from Elsevier, and Images 4D from [22] by permission from Macmillan Publishers Ltd. Scale bars 50 nm.

He and coworkers [21] investigated the desmosomal cadherin interaction in epidermis from newborn mice. The epidermis was processed by high pressure freezing followed by freeze substitution and embedding at room temperature in epoxy resin. Ultrathin sections were cut and stained with the conventional lead and uranium stains. Tomograms were collected by tilting about two orthogonal axes and tomograms were generated with isotropic resolution of approximately 2 nm. The authors' observed finger-like projections spanning across the intercellular space (28 nm wide) forming a dense mid-line believed to correspond to the area of cadherin associations (Figure 4(a)). These finger-like projections were tracked through the 3D volume and were found to be arranged in an irregular manner with tip-to-tip interaction only (Figure 4(b)i). These observations are inconsistent with the interdigitation model, though bore some resemblance to the molecular packing observed in the x-ray crystal of C-cadherin. In particular, the curved nature of the cadherin molecule seen by electron tomography did resemble C-cadherin ectodomains [50]. Since C-cadherins have 30–35% sequence identities to those of desmosomal cadherin ectodomains [59] the C-cadherin crystal structure was fitted into individual densities of cadherins to follow their interactions. Three distinct geometries were found resembling the letters W (Figure 4(b) ii), S (Figure 4(b) iii), and the greek letter *λ* (Figure 4(b) iv). Trans interactions occurred in W (23%) and S (43%) shaped cadherins pairs, whereas the addition of a third molecule with cis interactions produced a *λ* shape (40%) (Figure 4(b) v). The W shape closely resembled the two-fold symmetric strand dimer observed in the x-ray structure, which creates an exclusive molecular pairing due to the mutual binding of Trp^2^ side chains to the hydrophobic pocket of the dimeric partner. However, the *S* and *λ* shapes distorted this symmetry to allow nonsymmetric interactions amongst larger groups of molecules. In particular, the S-shape also formed trans interactions and was related to the W-shape by rotation of the lower molecule relative to the upper one by 90°. This rotation had the effect of pulling one of the Trp^2^ side chains out of its neighbors hydrophobic pocket, thereby making available for binding of a third molecule. Addition of this third molecule produced the *λ*-shape, which used the free Trp^2^ to form a cis interaction. Interestingly this strategy of adding additional molecules in both cis and trans appeared to generate interacting networks of up to 6 molecules (Figure 4 vi). In such networks, a stochastic arrangement of cadherins interwoven at the midline formed a tangle of molecules (Figure 4 vi). Despite the distortion of the strand dimer interface seen in the x-ray structure, the authors concluded Trp^2^ insertion into the hydrophilic pocket of a neighboring molecule was responsible for many of the interactions within the network. Such interactions were possible because of flexibility in the peptide linking Trp^2^ to the EC1 domain, and to potential flexibility between successive EC2–EC5 domains. Calcium binding at the EC domain linkers is likely to control the overall flexibility of the cadherin molecule and perhaps to control the propensity of these various observed shapes. Flexibility was also observed in the orientation of the EC5 domain relative to the membrane. These EC5 domains were observed to form pairs or triplets with other EC5 domains. Interestingly, the molecules involved in EC5 groups near the membrane were engaged with different molecules at the midline, providing a mechanism for propagating interactions along the plane of the membrane. In summary, this investigation stressed the crucial role of Trp^2^ and its hydrophobic pocket suggesting that Trp^2^ can mediate both cis and trans interactions. Such interactions are possible due to the flexibility of the cadherin maximizing interactions between cadherin forming the junctions. As a result the tangle arrangement of cadherins at the midline may form physical bonds between cells whereas the intracellular domain may be responsible for the lateral stabilization of the groups.

A new model of cadherin associations in 2D by Al-Amoudi et al. [65] contradicted the irregular or stochastic cadherin associations observed by He et al. [21]. Desmosomes from human epidermis were imaged by CEMOVIS. The extracellular domains of desmosomal cadherins were visualized as densely packed and periodically arranged but protruding in a straight manner from the opposing cell membranes (Figure 4(c)). Cadherin periodicity at the dense mid-line was 5 nm within the 33 nm wide ECD. Differences in cadherin associations were attributed to the deleterious effect of conventional specimen preparation, citing that aggregation forces during dehydration may have caused the cadherins to form the observed stochastic associations [66]. However, such conclusions were derived from a single 2D projection and from a preparation technique, which is known to create sections that have been compressed between 30–45% along the cutting direction [67].

Recently, a 3D model of the human epidermis desmosome was presented by Al-Amoudi et al. [22]. The epidermis was processed by CEMOVIS and ultrathin cryosections of tissue were then imaged in low dose mode. Tomograms were generated by tilting about a single axis, thus producing an anisotropic resolution of approximately 3.4 nm. The study verified the periodic arrangement of the cadherins observed in 2D but at ~7 nm intervals along the mid-line rather than the 5 nm previously reported. In 3D, these periodic array of densities were curved (at an angle of 20° with the cell membrane), resembling the X-ray structure of C-cadherin and adopting an organization with alternating trans and cis interactions. The authors state that the highly conserved Trp^2^ was most likely involved in adhesive binding and two distinct geometries were seen. The W-shape (as described in the He et al. model [21]) formed the trans interactions between molecules emanating from opposing cell surfaces. However, differing from the He et al. model [21] V-shape interactions between molecules emanating from the same cell membrane formed the cis interactions. These interactions were likely to be restricted to the EC1 domain when their concave surfaces faced each other. Such arrangement at the midline formed alternate cis and trans dimers resulting in a highly packed regular zipper-like organization (Figure 4(d) i–iii).

**Figure 5:**
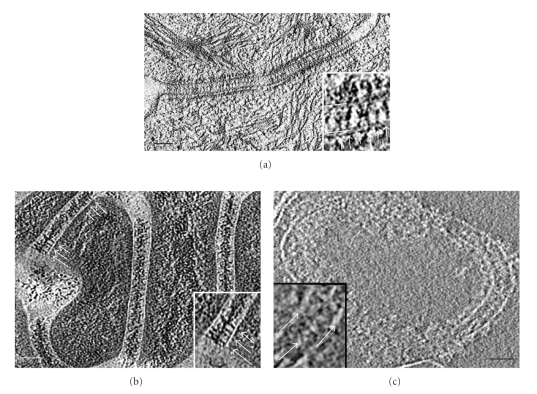
Electron tomography of desmosomes from freeze-substituted resin-embedded epidermis of newborn mouse (a). Cadherin densities can be seen spanning the extracellular space and individual intermediate filaments projecting towards the cell membrane in the cytoplasm. The inset provides a magnified view of the irregular nature of cadherin association creating the dense mid-line. (b) In the cow snout *stratum spinosium*, cadherin densities can be seen spanning the extracellular space but the density of cadherin molecules is appreciably much higher. The dense mid-line can be observed by viewing the slice at a glancing angle however, in some areas, the irregular nature of cadherin association is visible (arrows) and can be seen magnified in the inset. Membranes are highlighted by lines and the cadherins by arrows in both a and b. (c) Cryoelectron tomography of isolated desmosomes in the frozen unstained state. A slice from a tomogram showing a desmosome which has curled up at the edge to provide a direct view of cadherin organisation. The two bilayers are clearly visible and a midline composed of globular densities can be seen bisecting the extracellular space (inset). These globular densities (arrow) probably correspond to the groups of cadherin molecules seen previously in freeze-substituted samples. Reproduced with permission from, [23] the Biochemical Society (http://www.biochemsoctrans.org/). Scale bars 50 nm.

Of the two 3D models that exist describing cadherin interactions, they differ in that one describes the cadherin interaction with periodicity and the other with irregularity. Explanations for the disparity in these models are that cadherin packing varies in different tissue or that the preparative methodologies affect the results. A study by Owen et al. [23] attempted to evaluate the effects of the preparation methods on cadherin interactions in two types of tissues from two different species in 3D. In this study high pressure frozen, freeze substituted epoxy resin ultrathin sections of neonatal mouse epithelium and the well-characterized bovine snout *stratum spinosium* were first compared with electron tomography. Desmosome structure displayed similar features in both samples although the densities of cadherins and IF were much lower in the mouse epithelium (Figure 5(a)) compared to bovine snout (Figure 5(b)). A remarkable difference in ECD distances was seen between both tissues. Mouse skin ECD distances of 32.70 ± 1.97 nm (*n* = 4) confirmed existing results by He et al. [21], whereas the bovine snout ECD distances of 42.7 ± 2.63 nm (*n* = 8) were notably larger than the mouse. In order to compare the effects of preparation methods on the desmosome structure, purified isolated desmosomes from bovine snout were used as the sample because of their well-documented biochemical constitution [68, 69] and because milligram quantities of desmosomes cores were readily isolated, consisting of their plasma membrane and intracellular components as well as some intracellular filamentous material. For tomography, isolated desmosomes were vitrified in suspension by plunge freezing and imaged at low temperature in low-dose mode. In this way the need for sectioning was unnecessary therefore eliminating any possibility of compression artifacts that may have been caused by sectioning. In 3D the basic trilaminar features as seen in stained desmosomes *in situ* were observed that is, apposed membranes separated by a distinct midline and flanked by remnants of intracellular material. Irregular patterns of globular densities within the intracellular space were consistent with the presence of unresolved groups of cadherin molecules along the mid-line (Figure 5(c)). Such irregular groupings of cadherin molecules were observed previously in freeze substituted mouse epidermis. Therefore, this study suggested that the protocol used for freeze substitution did not cause major rearrangement of cadherin packing within the intercellular region. The authors hypothesized that, if any possibility of compression artifacts were eliminated then, the differences reported between cadherin associations in freeze substituted resin-embedded new-born mouse epidermis and frozen hydrated sections of human forearm epidermis might be due to different mechanical conditions experienced by the respected tissues. In tissues placed under repeated, directional stress, irregular groups of cadherin molecules might be remodeled to form a quasi-ordered array to provide the greatest directional adhesive strength.

## 5. Conclusion, Perspectives, and Prospects for the Future

Desmosomes have an intrinsic mechanical role in maintaining the integrity of epithelium and this characteristic has been well documented. The desmosome also plays a role in directing tissue morphogenesis, sensing environmental signals, regulating tissue homeostasis, and as being a phenotypic determinator. On a structural level, these processes are controlled by the interconnection of the proteins composing the desmosome. Of particular interest is how desmosomal cadherin isoforms contribute to tissue morphogenesis and phenotypic determination. These processes are almost certainly controlled by intercellular interactions between desmosomal cadherins, which has been the topic of this review. In particular, cadherin interaction models using isolated cadherin domains have provided evidence for various modes of cadherin associations. In electron tomograms of desmosomes from both mouse skin and human skin samples, the highly conserved Trp^2^ and its hydrophobic pocket has been deemed the most likely mechanism of adhesive binding. Neither structure shows any evidence for the interdigitation model proposed on the basis of intermolecular force measurements. However, these two models differ in that one observed cadherin interactions with periodicity and the other with irregularity. Although differences in preparation methods may have contributed to the observed differences, a study comparing freeze substituted samples with native vitrified samples of bovine snout desmosomes suggested that freeze substitution is capable of preserving molecular detail in the tissue. Another explanation for the structural differences may be the different mechanical conditions experienced in the various tissues: newborn mouse skin, cow snout, and the human forearm skin. The ~30% increase in Dsg-3 and Dsc-3 expression in thick versus thin skin may well signify how differing mechanical differences can affect desmosome composition [70, 71] and, indirectly, cadherin association patterns. Therefore, further analyses of desmosomal cadherin associations are warranted to unequivocally demonstrate the adaptive response of desmosomal cadherin associations to mechanical stress. Indeed, although the basic organization of desmosomes is conserved across different species and tissues, they differ in their polypeptide composition [72, 73]. In fact, variation in desmosome frequency, diameter [74] and ECD thickness [75] have all been observed within a given tissue. Distinct expression profiles of cadherins across the epidermis is also well documented [71]. Such changes are thought to reflect the adhesive requirements of the epidermis as stratification proceeds [70], but might also reflect that desmosomes are less dynamic and able to form a more stable structure whilst allowing keratinocytes to maintain their adhesive properties [76, 77]. Understanding the effects of differentiation and mechanical shear on the structural associations within desmosomes represents a challenging, but important task for future investigations.
